# Exploring socio-contextual factors associated with male smoker’s intention to quit smoking

**DOI:** 10.1186/s12889-016-3054-5

**Published:** 2016-05-13

**Authors:** Minsoo Jung

**Affiliations:** Department of Health Science, Dongduk Women’s University, 23-1 Wolgok-dong, Seongbuk-gu, Seoul 136-714 South Korea

**Keywords:** Quit smoking, Health communication, Media exposure, Socioeconomic position

## Abstract

**Background:**

Programs to encourage smokers to quit smoking tobacco have been implemented worldwide and are generally viewed as an effective public health intervention program. However, few studies have examined the social factors that influence a smoker’s intention to quit smoking. This study investigated the socio-contextual factors that are associated with the intention to quit smoking among male smokers in South Korea.

**Methods:**

Data were obtained from a 2014 nationally representative panel that examined the influences of mass media on the health of the Korean population. Members of this panel were recruited using a mixed-method sampling and a combination of random digit dial and address-based sampling designs. Survey questions were based on those used in previous studies that assessed the effects of social context, including mass media and social capital, on health. Multivariate logistic regression analyses of the answers of 313 male smokers were undertaken.

**Results:**

Male smokers who participated in community-based activities were 2.45 times more likely to intend to quit smoking compared to male smokers in general (95 % confidence interval [CI]: 1.25–6.82). In addition, male smokers who participated in informal social gathering networks were 2.38 times more likely to intend to quit smoking compared to male smokers in general (95 % CI: 1.11–5.10). Moreover, male smokers with high smartphone use were 1.93 times more likely than smokers with low smartphone use to intend to quit smoking within one year (95 % CI: 1.07–3.46).

**Conclusions:**

A supportive environment that enables male smokers to access beneficial health information and that encourages them to quit smoking is necessary for a stop-smoking program to be effective. The result of this study contribute to establishing a new smoking control policy by identifying socio-contextual factors related to the intention to quit smoking.

## Background

Many developed countries have implemented multifaceted anti-smoking policies in the twenty-first century that include price- and non-price-based approaches to lowering tobacco smoking (henceforth, smoking) rates [1]. The decrease in smoking rate in many developed countries has slowed down, and the changes in smoking rate in some countries are stagnant [2]. In particular, a high smoking rate among male smokers is a reason for the stable prevalence of adult smoking in Asian countries. In 2012, adult smoking rates in China, Japan and South Korea (henceforth, Korea) were 49.0, 36.6 and 43.7 %, respectively [3]. The smoking rate among adult Korean males in 2008 was 40.9 % and was 43.1 % 2013 [4]. Therefore, the change smoking rate in Korea has stagnated, and encouraging smoking cessation in male smokers is important.

Price-based policies have been implemented in Korea eight times between 1986, when tobacco advertising was restricted through the Tobacco Business Act, and December 2014 [5]. Since 2005, approximately 253 healthcare centers in Korea have been operating smoking cessation clinics. In addition, local governments have discouraged smoking by establishing community level smoke-free regulations and declaring smokeless areas [5]. The use of graphic warning labels, which were first introduced in Canada in 2001 and are now used globally [6], was approved by Korean National Assembly standing committee in accordance with the World Health Organization Framework Convention on Tobacco Control. Regardless, Korea, with the highest smoking rate among Organization for Economic Co-operation and Development countries, is attempting to develop more effective anti-smoking policies. Other East-Asia countries, such as China and Japan, have a similar problem with stagnant smoking rates, which has been attributed to their similar societal, behavioral and cultural views on smoking [7, 8].

The trans-theoretical model (TTM) regarding behavioral change can be used to explain the process of change that smokers go through to succeed in quitting smoking. Such modelling has been widely used to investigate change-positive behaviors [9]. According to the TTM, a smoker goes through pre-contemplation, contemplation, preparation, action and maintenance stages when quitting smoking [10, 11]. A critical factor in attaining these stages of behavioral change is the smoker’s intention to quit smoking [12–15]. This is because the TTM is a helical process with the capacity for repeated entrance and regression rather than a process with unidirectional progression. Among the TTM constructs, the intention to quit smoking before quitting has been suggested to be a predictor of whether the smoker will participate in a smoking cessation program, attempt to quit smoking and succeed in quitting [14, 16, 17]. Therefore, when developing various quitting-smoking programs, developed countries have conducted studies on socio-demographic and smoking-related factors that affect participants’ intentions, attempts and successes when quitting smoking [15, 18].

However, smokers in the preparatory stage of a behavioral change do not easily show a behavioral change despite having a greater level of intention that that of smokers in the contemplation stage [19]. Therefore, the main factors affecting the intention to quit smoking need to be determined for smokers with little or no intention to quit. Nevertheless, many preceding studies on smoking cessation described the cognitive factors of smokers in different behavioral change stages than those in the TTM model [13, 14, 17, 20]. These studies described general, smoking-related and health-related characteristics of smokers during different behavioral change stages [21]. Few studies have reported on factors that influence the intention to quit smoking or whether the attempts were successful [18, 19, 22]. In these studies, the subjects were small groups of young adults, office workers and participants in various healthcare center smoking cessation programs. Hence, the study designs were unsuitable for a nationwide assessment of the various contextual factors that affect a male smoker’s intention to quit smoking. This nationwide study examined socio-contextual factors regarding the intention to quit smoking in adult male smokers. We focused on the intention to quit smoking but our TTM model also included several other variables.

## Methods

### Study participants

The data for this study came from a nationally representative sample of Korean adults who participated in Hankook Research’s Master Sample Panel©. Members of this panel were recruited using a mixed-method sampling frame and a combination of random digit dial and address-based sampling designs, which allows sampling of individuals who do not have a telephone land line. Based on the purpose of this study, male non-smokers and all females were excluded; only male smokers were included (*n* = 313). A survey questionnaire was provided online to participating male smokers. Participants received a nominal cash incentive to participate in the survey, and the final response rate was 87.0 %. Survey questions with missing values for key analytical variables were excluded using a pairwise method.

### Ethics statement

Approval for the study was granted by the Korea National Institute for Bioethics Policy Institutional Review Board (April 11, 2014; P01-201404-SB-19-00). All participants gave written informed consent to participate. The Ethics Committees of the Demographic Health Survey approved this consent procedure. Any information that could distinguish individual participants was not collected during the data collection process.

### Measures

The survey questions were based on those used in previous reports on the effects of social context, including mass media use and social capital, on health [2, 10, 20, 22, 23]. The questionnaire included topics adapted from the Health Information National Trends Survey (http://hints.cancer.gov/).

#### Dependent variables

This study focused on current male smokers regardless of numbers of cigarettes consumed per day. The outcome variable was the intention of the participant to quit smoking. All self-rated smokers were asked to report their intention to quit smoking on a 5-point scale: (1) “Intends to take action within the next 30 days,” (2) “Intends to take action within the next 90 days,” (3) “Intends to take action within the next 6 months,” (4) “Intends to take action within the next 12 months,” and (5) “Has no intention to take action within the next 12 months.” The initial question was “Do you have an intention to quit smoking?” The answers were grouped into two categories. Participants responding with (5) were coded as 0 and grouped as no intention to quit smoking. Those responding with (1–4) were coded as 1 and were grouped as intending to quit smoking.

#### Independent variables

Participant’s socio-economic position was based on education and annual household income levels. Participants were asked to identify their highest education degree earned: high school diploma or less; college degree; or postgraduate degree. Participants were asked to report their total annual household income before taxes: < USD 20,000; USD 20,000–39,999; USD 40,000–59,999; USD 60,000–79,999; and USD ≥ 80,000. General mass media usage was assessed by the following questions: “In the past 7 days, how many hours do you: watch television per day on average; listen to the radio; read a newspaper; search information with a smartphone; and read news on the Internet with a personal computer?” The response range was 0 to ≥ 5 h. We asked seven questions about barriers to finding desired health-related information. For each question, participants were asked to note whether there was a large problem, small problem or no problem at all when trying to obtain the desired information about their health.

We conducted a principal components analysis (PCA) to reduce the number of study variables and detect the internal variance structure in the relationships between variables. The PCA factors used to construct the information barrier index presented eigenvalues > 1 and factor loadings > 0.40. The first factor, “information access barriers”, accounted for 32.4 % of total variance (Cronbach’s alpha = 0.73). This factor included three of the seven barriers: “access to the Internet; difficulties using an on-line search tool or software; and the available information used too many technical terms”. A second factor, “information utilization barriers”, explained 26.4 % of total variance. This factor included the remaining four potential barriers: too much information; no way to tell if the information was accurate; no way to tell if the information was up-to-date; and no way to tell if the information was relevant to my situation. These factors were used to define a reverse scale for accessing individual capacity to access health information. A low alpha value of 0.5 indicated that the question did not have a unidimensional scale. In this study, an alpha value of 0.7 indicated that the question was acceptable for use as a measurement scale [24].

#### Potential confounders

Participants were asked to rate their own general health status on a 5-point Likert-type scale ranging from very good to very bad. The initial question was “How is your health in general?” The answers were eventually grouped into two categories. Participants reporting “very bad,” “bad,” or “average” for self-rated health (SRH) status were coded as 0 and were placed in the low-SRH group, whereas those reporting “very good” or “good” SRH status were coded as 1 and were placed in the high-SRH group. We also asked about outpatient visits, including dental and oriental medicine treatments during the month preceding the questionnaire. The responses were grouped into two categories of yes or no. In addition, we asked seven questions related to social capital to determine the degree of community social cohesion. Social capital included social structural features, such as interpersonal trust and norms of reciprocity and mutual aid that act as resources for individuals and facilitate collective social action [25]. Social capital thus forms a subset of the features of social cohesion. Participants were asked to note whether they “actively participated, participated, just joined or not involved at all in social gatherings in their community or by personal membership” for each social issue. After conducting the PCA, we detected two factors associated with the greatest proportion of the total variance. The first factor, “community-based activities”, accounted for 28.8 % of total variance (Cronbach’s alpha = 0.70) and included four community activities: workplace, neighborhood, volunteer and political activities. The second factor, “informal social gathering”, was associated with 22.5 % of total variance (Cronbach’s alpha = 0.64). This factor included the remaining three community activities: informal associations, get-together club and religious group activities.

### Statistical analyses

First, descriptive statistics and frequencies were derived for all variables. Second, bivariate analyses were undertaken for each potential predictor variable to identify socio-contextual factors associated with the intention to quit smoking. Barriers to obtaining health-related information or social capital were subjected to factor analyses. Subsequently, differences in the social characteristics between the groups with and without intentions to quit smoking were assessed by the chi-square test. Finally, multivariate logistic regression analyses were used to analyze potentially influential factors related to the intention of adult Korean males to quit smoking. All analyses were conducted using SPSS v.21.0 software (IBM SPSS, Armonk, NY, USA).

## Results

### General sample characteristics

Of the 313 male smoker participants, 81 (25.9 %) were in their 40s and 33 (10.5 %) were ≥ 60 years of age (Table 1). A total of 214 participants (68.4 %) had a college or post-graduate degree, and approximately 35.1 % of all participants earned USD 20,000–USD 40,000 per year. A total of 264 participants (84.3 %) reported high SRH status. With regard to medical utilization and mass media use, 152 participants (48.6 %) had received outpatient care during the month previous to completing the questionnaire, and the two most frequently used sources of mass media were television and smartphone. A total of 215 (68.7 %) participants watched television ≥ 1 h per day, and 125 (40.0 %) searched for information via a smartphone. However, only 65 (20.8 %) read newspapers for ≥ 30 min per day. Seventh-two (23.0 %) participants expressed an intention to quit smoking, and 241 (77 %) had no intention to quit smoking.

**Table 1:** General characteristics of the sample, 2014 (male smokers, *n* = 313)

	*n*	%		*n*	%
Age			Television Watching		
20–29	55	17.6	30 min or less	51	16.3
30–39	89	28.4	30 min to one hour	47	15.0
40–49	81	25.9	One hour to two hour	102	32.6
50–59	55	17.6	Two hour to three hour	65	20.8
60 or older	33	10.5	Three hour or more	48	15.3
Education			Radio Listening		
high school or less	99	31.6	No listening	121	38.7
college	178	56.9	10 min or less	41	13.1
post-graduate	36	11.5	10 min to 30 min	55	17.6
Income			30 min to one hour	48	15.3
US$20 K or less	35	11.2	One hour or more	48	15.3
US$20 K–40 K	110	35.1	Newspaper Reading		
US$40 K–60 K	97	31.0	No reading	143	45.7
US$60 K–80 K	45	14.4	10 min or less	46	14.7
US$80 K or more	26	8.3	10 min to 30 min	59	18.8
SRH			30 min or more	65	20.8
High SRH	264	84.3	Smartphone Browsing		
Low SRH	49	15.7	no use or less than 10 min	43	13.7
Medical Utilization			10 min to 30 min	51	16.3
Never	161	51.4	30 min to one hour	94	30.0
One time	86	27.5	One hour to two hour	81	25.9
Two time	35	11.2	Two hour or more	44	14.1
Three time or more	31	9.9	Computer Searching		
Intention to quit smoking			No use or less than 10 min	41	13.1
No intention to take action within the next 12 months	72	23.0	10 min to 30 min	36	11.5
Intends to take action within the next 12 months	136	43.5	30 min to one hour	59	18.8
Intends to take action within the next 6 months	68	21.7	One hour to two hour	102	32.6
Intends to take action within the next 90 days	14	4.5	Two hour to three hour	40	12.8
Intends to take action within the next 30 days	23	7.3	Three hour or more	35	11.2
Total	313	100.0		313	100.0

### Differences between the groups with and without an intention to quit smoking

The group intending to quit smoking had more participants with a high educational level (*p* < 0.05) and more participants who had received medical service compared to those who did not intend to quit smoking (*p* < 0.01, Table 2). More participants who intended to quit smoking read a newspaper than those who did not (*p* < 0.01).

**Table 2:** Bivariate analyses of the differences between the smoking and quit smoking groups, 2014

	Smoking (*n* = 208)	Intention to quit smoking (*n* = 105)	*p*-value		Smoking (*n* = 208)	Intention to quit smoking (*n* = 105)	*p*-value
Age				Television Watching			
20–29	63.6	36.4	ns	30 min or less	66.7	33.3	ns
30–39	67.4	32.6		30 min to one hour	66.0	34.0	
40–49	61.7	38.3		one hour to two hour	68.6	31.4	
50–59	72.7	27.3		two hour to three hour	67.7	32.3	
60 or older	69.7	30.3		three hour or more	60.4	39.6	
Education				Radio Listening			
High school or less	76.8	23.2	<0.05	no listening	74.4	25.6	ns
College	61.2	38.8		10 min or less	65.9	34.1	
Post-graduate	63.9	36.1		10 min to 30 min	63.6	36.4	
Income				30 min to one hour	58.3	41.7	
US$20 K or less	74.3	25.7	ns	one hour or more	58.3	41.7	
US$20 K–40 K	66.4	33.6		Newspaper Reading			
US$40 K–60 K	68.0	32.0		no reading	76.2	23.8	<0.01
US$60 K–80 K	68.9	31.1		10 min or less	60.9	39.1	
US$80 K or more	46.2	53.8		10 min to 30 min	54.2	45.8	
SRH				30 min or more	60.0	40.0	
High SRH	65.9	34.1	ns	Smartphone Browsing			
Low SRH	69.4	30.6		no use or less than 10 min	79.1	20.9	ns
Medical Utilization				10 min to 30 min	70.6	29.4	
Never	72.0	28.0	<0.01	30 min to one hour	60.6	39.4	
One time	68.6	31.4		one hour to two hour	61.7	38.3	
Two time	45.7	54.3		two hour or more	70.5	29.5	
Three time or more	54.8	45.2		Computer Searching			
				no use or less than 10 min	78.0	22.0	ns
				10 min to 30 min	75.0	25.0	
				30 min to one hour	59.3	40.7	
				one hour to two hour	64.7	35.3	
				two hour to three hour	65.0	35.0	
				three hour or more	62.9	37.1	

*ns* not significant

### Socio-contextual factors of the intention of smokers to quit smoking

After controlling for potential covariates, male smokers ≥ 60 were 1.90 times more likely to intend to quit smoking than young male smokers (95 % confidence interval [CI]: 1.06–3.34) (Table 3). Males who participated in community-based activities were 2.45 times more likely to intend to quit smoking than male smokers who did not participate in community-based activities (95 % CI: 1.25–6.82). Similarly, males who had informal social gathering networks were 2.38 times more likely to intend to quit smoking than males who did not participate in formal social gathering networks (95 % CI: 1.11–5.10). No differences in information utilization or access were observed between the groups with and without the intention to quit smoking. However, mass media usage was associated with smokers who intended to quit smoking. After controlling for significant covariates, male smokers who used a smartphone were 1.93 times more likely to have an intention to quit smoking than male smokers who did not use a smartphone (95 % CI: 1.07–3.46).

**Table 3:** Adjusted odds ratio and 95 % confidence intervals for male smokers who intended to quit smoking within a year, 2014 (*n* = 313)

		aOR	95 % CI		*p*-value
			Lower	Upper	
Socioeconomic Position	Education	1.706	0.574	5.068	ns
	Income	1.201	0.715	2.017	ns
Health Status	Age	1.885	1.062	3.343	<0.05
	SRH (Ref. = high)	1.000			
	low status	0.593	0.142	2.479	ns
	Medical Utilization (Ref. = none)	1.000			
	received outpatient care in the last month	1.090	0.596	1.991	ns
Social Capital	Community-based Activities	2.453	1.249	6.823	<0.01
	Informal Social Gathering	2.378	1.110	5.096	<0.05
Information-Seeking Capacity	Utilization Capacity	0.811	0.426	1.544	ns
	Access Capacity	0.677	0.305	1.502	ns
Media Use	Television	0.684	0.396	1.180	ns
	Radio	0.923	0.606	1.404	ns
	Newspaper	0.965	0.579	1.610	ns
	Smartphone	1.927	1.074	3.458	<0.05
	Computer	0.947	0.595	1.507	ns
NagelKerke R^2^		0.406			

Notes: The dependent variable is male smokers who intended to quit smoking within a year: Yes (1) and No (0)

All models are additionally adjusted for sampling region

*ns* not significant

## Discussion

Programs to encourage smokers to quit smoking tobacco have been implemented worldwide, and are generally viewed as an effective form of public health intervention. However, few studies have examined the socio-contextual factors that affect the intention to quit smoking in adult male smokers. Our results show that Korean adult male smokers who intended to quit smoking have significantly higher levels of social capital and smartphone use than those who did not. Three implications can be drawn from our results.

First, this study shows that a supportive socio-contextual environment, which enables male smokers to access beneficial health information as support while they quit smoking, may be necessary when trying to increase the effectiveness of a smoking cessation program at an individual-smoker level. An individual’s intention in the field of health education is important to produce a behavioral change [26, 27]. Although it is possible that, smokers reach out to new networks to get away from their currently smoking peers when intending to quit it is also possible that they weigh the benefits and costs of stopping smoking to evaluate whether their situation is favorable for quitting smoking [11, 27]. The TTM approach attempts to describe an individuals’ processes and stages of change associated with behavioral modifications and has been successfully applied in studies of various preventive behaviors [28, 29]. Our TTM results suggest that supportive socio-contextual factors, such as access to health information and beneficial relationships, help to smokers and such factors should be included when applying the TTM approach [30].

Second, the observation that the availability of social capital or a smartphone can affect intention to quit smoking indicates that smoking behavior can be changed through social relationships. As smokers’ exposure to health information through social networks or digital devices increases, they have an increasing chance of adopting healthy behaviors [24, 31]. Thus, as more friends and acquaintances of smokers stop smoking, the smokers have a greater chance to stop smoking [32]. Social relationships have a strong influence on substance use, and it is well documented that smokers tend to form social relationships with other smokers [33]. In addition, smoking behaviors are significantly associated with socio-economic indicators; for example, it is more difficult to encourage smokers in a low-income bracket to quit smoking than to encourage high-income bracket smokers to quit. However, smoker’s beliefs and attitudes towards specific health issues can be changed through their exposure to mass media [34]. The results of this study suggest a benefit to establishing a smoking control policy that would supply social network services at an interpersonal level.

Third, governments may need to publish and distribute health information through social networks regarding the risks of smoking, as a simple abundance of social capital is insufficient for an effective smoking cessation program [35]. Our results suggest a need to include social capital when developing smoking cessation programs at the community level. Harmful or beneficial health behaviors can spread to others, and the range of such proliferation can be substantial [35–37]. Smoking has long been a topic of social controversy over the health risks of tobacco to nonsmokers among a smoker’s peers, including secondhand smoke, which is becoming undesirable. When one person stops smoking, a ripple effect can influence the behavior of friends, friends of those friends and so on [36, 37]. In addition, the ripple effect becomes more influential when the group’s level of social cohesion is high [38]. Our results show that male smokers who participated in community-based activities were more likely to intend to quit smoking compared to make smokers in general. In addition, male smokers who participated in informal social gathering networks were more likely to intend to quit smoking compared to male smokers in general. These results suggest that community-based organizations provide support to individuals that would like easy access to information on stopping smoking, particularly via the provision of strategic media advocacy [39].

Several study limitations should be noted. First, a cross-sectional dataset was used; thus, caution is advised when examining potentially causal relationships. Second, not all TTM components were applied in our analysis because our use of the TTM was restricted to health communication factors. Third, the possible presence of heterogeneity in our sample of those who intended to quit smoking needs to be considered, particularly given that those who intended to quit in the next 30 days were grouped with those who intended to quit in the next 12 months. We manipulated the outcome variables in an attempt to consider this heterogeneity; however, the potential effect of such heterogeneity could not be assessed due to the small sample size in our study. The causal relationships inherent in quitting smoking need to be investigated to reduce these limitations. A longitudinal study based on a worldwide national sample is warranted to determine the applicability of our results to other nations. There is also a need to determine the applicability of the present findings to female smokers, both in Korea and abroad.

Despite these limitations, the current study revealed several main factors that affect the intention to quit smoking among adult male smokers who reported little or no intention to quit smoking. Personalized smoking cessation and intervention programs that incorporate socio-contextual factors should be developed. Such programs should assist those intending to quit smoking, help them progress through the stages of quitting smoking, and help those who have just quit smoking overcome abstinence symptoms and enter the nonsmoking maintenance stage.

## Conclusions

Korea adopted a powerful smoking control policy in 2015 that included a significant price increase for tobacco products. The cost of tobacco in Korea had not increased for over a decade. Consequently, many smokers have sincerely considered quitting smoking. Nonetheless, few studies have been conducted to determine the variables that influence a smoker’s intention to quit smoking. This study identified socio-contextual factors affecting the intention to quit smoking and may assist in establishing a new smoking control policy. Moreover, our results may influence the design of follow-up studies related to preventing diseases associated with smoking, such as lung cancer and chronic obstructive pulmonary disease.

### Ethics

Approval for the study was granted by the Korea National Institute for Bioethics Policy Institutional Review Board (April 11, 2014; P01-201404-SB-19-00). All participants gave written informed consent to participate. The Ethics Committees of the Demographic Health Survey approved this consent procedure. Any information that could distinguish individual participants was not collected during the data collection process.

### Consent to publish

Not applicable.

### Availability of data and materials

Data sharing: Participant level data are available from the corresponding author.

## Abbreviations

FCTC: framework convention on tobacco control
GWL: graphic warning labels
OECD: Organization for Economic Co-operation and Development
SEP: socio-economic position
SRH: self-rated health
TTM: trans-theoretical model
